# The Teeth of Deaf Mutes

**Published:** 1885-08

**Authors:** J. Howard Reed


### THE TEETH OF DEAF MUTES.

BY J. HOWARD REED, D. D. S., M. D. S.

Deaf mutes are found in all the walks of life. They number in their community skilled artisans, artists of pen and pencil, editors and poets of no mean reputation; and it may be of interest to know that the ball-lock device on pocket-books was invented by one of their number, and several bright minds are following professional pursuits. The immediate cause of congenital deafness is malformation, misplacement, a faulty arrangement, or want of the hearing organs; lack of constructing material, a thickening of the membranes, or the inner chamber of the ear being filled with mucous or bloody matter instead of the limpid fluid which ordinarily exists there, also rank as causes. Accidental deafness may be caused by mechanical or local means. But the most deadly enemy to the hearing organs is fever, in its various forms. About one-half of the cases of accidental deafness are caused by scarlet fever. This class of diseases produces deafness by inflammatory action upon the auditory nerves and that portion of the brain to which they are attached, ending in paralysis or insensibility of the nerve to sound. Mental trouble may affect the intellectual brightness of a person without in the least affecting his hearing.

Studying the antecedents of deaf mutes, three facts are of note; first, that the largest number of congenital deaf mute children are born of deaf mute parents, or about ten per cent. of the births under the named circumstances are deaf mutes; second, that the next largest number of congenital deaf mute births come from a blood marriage of the parents; third, that the third largest number come from one of the contracting parties to a marriage being a deaf mute, and in this latter case there is only a slight tendency for its transmission. These people possess quick and violent tempers, and show the traits of jealousy and sensitiveness, determined, however, according to the refinement of their surroundings. These traits are not an abnormality, but naturally arise from the fact that they are a people who are seldom sought after by the hearing and speaking.

The first thing in history relating to the teeth of deaf mutes that it has been my fortune to come across, is that of the case of James Mitchell, a boy congenitally deaf, dumb and blind; the only sense of sound that he could recognize was that conveyed to him through the medium of the teeth. The most noticeable circumstance of his childhood was his evident eagerness to strike upon his front teeth anything he could lay his hands on. This he would do for hours, and he seemed particularly gratified if the article was metal, or a hard substance that would give a sharp sound. His favorite play was to allow a key to vibrate between his teeth, suspended from a string. A musical snuff box being given him and placed between his teeth, seemed to afford him exquisite delight. When the notes would end he would still examine it with his tongue and fingers, with great curiosity. It is a common thing among deaf mutes to awaken the sensations through the vibrations of sound caused by metallic substances coming in contact with the teeth. Examining closely the dental characteristics of deaf mutes, there will be nothing that will pointedly strike you. The dark-bronze yellow tooth will always be associated with the pronouncedly bilious temperament. A bluish-white tooth will generally tell us the nervous person, and the merry red face and sanguine temperament have teeth of a cream color. Occasionally a departure from this rule will be found, and you may even see the characteristics of two or three temperaments represented in as many teeth. This latter abnormality, however, has no relation to the general color line of the teeth, which, on the average, is excellent. The quality of the teeth is slightly below the average, and those teeth, mainly, are affected only where the physical make-up is such as to encourage the ravages of decay. Take twenty per cent of the deaf mutes, and you will find some curious forms given to the teeth. One will present a mouth with the superior incisors perfect in every respect, and having the lower incisors perfect in color and shape, but dwarfed to one-third their normal size. A second patient will present with four stumps of centrals, and having the laterals towering up like guardian giants. In one or two mouths almost a perfect picket-rail fence has been seen, extending from the median line to as far as the first or second molars, with the cusps of the bicuspids and molars like a chalky formation. The certain scarlet-fever marks on the teeth cannot be mistaken; frequently the bicuspids will show the marks on the four or eight teeth, presenting that yellow or light brown color, much as if the tooth was entirely robbed of enamel. Some teeth look as if nature had declared perpetual warfare against syphilitic tendencies and inheritance, and the effects of scarlet fever on them. Three or four cases have been observed in which the six anterior teeth, both upper and under, presented the appearance of graduated steps that seemed almost mechanical in their precision. The only physical abnormality about a deaf mute is the lack of expression about the face, with a look of stolidness. The muscles of the facial region appear well developed, more particularly those of the eyes and mouth, as might be expected when it is remembered that they are put to constant exercise in the endeavor to convey their impression and give expression to thought. Peculiarities in the articulation are often present. Eruption is delayed on the average about two years in the bicuspids, and all the teeth are more or less delayed in their appearance. Decay of the teeth occurs, but it is more slow in its effects than in the teeth of the hearing and speaking. One thing will impress you upon examining the mouth of a deaf mute, and that is that either the fissures in the back teeth are ground perfectly smooth, are obliterated, or only slightly distinct, or the front teeth show exceeding great wear, and frequently the entire set of teeth is one continuous row of flat ivory. Irregularities do not occur in any larger percentage than in the teeth of children with all their faculties. Their teeth are very sensitive, apparently at the expense of their hearing powers. In artificial work it is necessary to study strength with the maximum of bulk, instead of strength with the minimum of bulk, as it is essential to have strength to withstand the great strain to which deaf mutes subject their teeth, and as the matter of space for the vocal organs need not be taken into account. To sum up, I have myself found deaf mutes anything but desirable as patients. I can perform all operations pertaining to dentistry upon them, but a certain tact and judgment is necessary, and all heroic treatment must be absolutely avoided.

